# Clinical results of image-guided interstitial brachytherapy with or without external beam radiotherapy for postsurgical vaginal recurrence of cervical and endometrial cancers

**DOI:** 10.1007/s11604-021-01229-y

**Published:** 2021-12-01

**Authors:** Ayae Kanemoto, Tadashi Sugita, Fumio Ayukawa, Kotaro Takahashi, Ayano Horiuchi, Kazufumi Haino, Akira Kikuchi

**Affiliations:** 1 Department of Radiation Oncology, Niigata Cancer Center Hospital, 2-15-3 Kawagishi-cho, Niigata, 951-8566 Japan; 2 Department of Gynecology, Niigata Cancer Center Hospital, Niigata, Japan

**Keywords:** Brachytherapy, Image-guided brachytherapy, Vaginal recurrence

## Abstract

**Purpose:**

This study aimed to evaluate the clinical outcome and efficacy of image-guided interstitial brachytherapy (ISBT) for postsurgical vaginal recurrence of cervical and endometrial cancers.

**Materials and methods:**

The study included 11 patients who received CT-based image-guided high-dose-rate ISBT with or without external beam radiotherapy (EBRT). Local control, progression-free survival, and treatment-related toxicities were evaluated retrospectively.

**Results:**

Of the 11 patients, 4 underwent ISBT with EBRT and the other 7 ISBT alone; two of the latter patients received previous pelvic radiotherapy. After a median follow-up of 43.9 months (range 3.9–92.7 months), the 2-year local control rate was 100%. The median equivalent doses in 2 Gy fractions received by at least 90% of the clinical target volume for ISBT with versus without EBRT were 82.2 Gy (range 60.4–84.2 Gy) versus 69.0 Gy (range 50.8–98.2 Gy). The 2-year progression-free survival rates after ISBT with versus without EBRT were 75% versus 80%, and the difference was not significant (*p* = 0.74). Grade 3 late toxicities occurred in two patients.

**Conclusion:**

Our radiotherapy strategy using image-guided ISBT, either with or without EBRT, for postsurgical vaginal recurrence showed effective treatment outcomes.

## Introduction

Radical treatment is often indicated for pelvic recurrence of cervical and endometrial cancers. However, radical surgical treatments, such as curative resection or pelvic exenteration, are difficult to perform because of the presence of several organs, such as the rectum, intestines, bladder, and urethra, near the recurrent lesion. On the other hand, pelvic recurrence, especially isolated vaginal recurrence, can be treated with curative salvage radiotherapy using brachytherapy (BT) [1–3]. The high-dose-rate (HDR)-BT dose can be calculated using CT or MRI, and image-guided BT is suggested to maximize disease control and reduce toxicity [4].

In 2013, image-guided HDR interstitial BT (ISBT) was introduced for treating vaginal recurrence at our hospital. The aim of this study was to analyze the clinical results of image-guided HDR ISBT for patients with isolated postsurgical vaginal recurrence of cervical and endometrial cancers, as an initial experience, and to evaluate the efficacy of the treatment.

## Materials and methods

We reviewed our institutional clinical database to identify women who received salvage BT for vaginal recurrence from May 2013, when image-guided BT was introduced at our hospital, to August 2017. A total of 11 patients with isolated postsurgical vaginal recurrence of cervical or endometrial cancer had received image-guided HDR ISBT with or without external beam radiotherapy (EBRT) during this period and were followed for at least 3 months after treatment until March 2021.

In the patients who did not receive previous pelvic radiotherapy, EBRT was administered before ISBT when the recurrent tumor was identified at the edge of the vaginal cuff and was suspected to involve microscopic invasion to paravaginal tissues, or if the recurrent tumor was too large to treat with ISBT alone. EBRT was performed using a three-dimensional conformal technique. EBRT was delivered to the pelvis using a 15-MV linear accelerator. The superior margin of the field was located at the top of the L4–5 or L5–S1 vertebrae, and the inferior margin was located at the inferior border of the obturator foramen. The lateral margin was 1.5 cm lateral to the widest margin of the bony pelvis. A radiation dose of 30 Gy in 15 fractions was administered to the pelvic field using a 4-field box technique, with 20 Gy in 10 fractions delivered to a 4-cm-wide central shielded (CS) field using the anterior–posterior/posterior–anterior parallel-opposed field technique.

HDR-BT was delivered using an interstitial technique with metallic applicators and a cylindrical applicator to adequately cover not only the gross tumor volume (GTV), but also from the ventral to distal sides of the vaginal cuff mucosa. The GTV was defined as the total vaginal recurrent tumor, which was defined based on clinical and imaging (MRI pretreatment and CT at treatment initiation) findings. The clinical target volume (CTV) was defined as the GTV plus the distal vaginal mucosa and vaginal cuff. Ir-192 was used as the treatment source. After implantation, CT-based treatment planning was performed using the BrachyVision™ system (Varian Medical Systems Inc., Palo Alto, CA, USA), and the dose was prescribed to the CTV. Generally, the goal of treatment planning was to deliver the prescription dose to ≥ 90% of the CTV and to 100% of the GTV. The prescription dose of ISBT with EBRT was 30 Gy delivered in five fractions, and that of ISBT alone was 36 Gy in six fractions or 42 Gy in seven fractions. Dose–volume histograms were determined for the CTV and organs at risk, including the rectum and bladder. The equivalent dose in 2 Gy fractions (EQD2) was calculated as the combined dose of EBRT (excluding the dose with CS) and ISBT using the linear-quadratic formula. The *α*/*β* ratio was 10 Gy for the tumor and 3 Gy for the organs at risk.

Data were collected from the patients after obtaining comprehensive consent and were analyzed retrospectively. This study has been approved by the institutional review board of our hospital. Local control (LC) and survival rates were estimated using the Kaplan–Meier method. Late treatment-related toxicities were assessed at follow-up visits using the National Cancer Institute Common Terminology Criteria for Adverse Events, version 4.0. We divided the patients into two groups according to the radiotherapy method (ISBT plus EBRT vs. ISBT alone), and progression-free survival (PFS) was compared between the groups using the log-rank test. Data analysis was performed using the Ekuseru–Toukei software package (version 2010; Social Survey Research Information Co., Ltd.). *P* values < 0.05 were considered significant.

## Results

The patient and tumor characteristics are summarized in Table 1. Of the 11 patients, 7 were treated with ISBT alone and 4 with a combination of EBRT and ISBT. Two patients received previous pelvic EBRT. The tumor site treated with ISBT plus EBRT was the upper one-third of the vagina in all cases. The median maximum tumor size was 26.5 mm (range 15–44 mm) treated with ISBT plus EBRT and 17 mm (range 11–32 mm) treated with ISBT alone.

**Table 1:** Patient and tumor characteristics with respect to each radiotherapy method

Patient no	Age (years)	Primary tumor	Initial FIGO stage	Histology	Tumor site	Maximum tumor size (mm)	Tumor thickness (mm)	The period from initial surgery to current radiotherapy (months)	Previous pelvic radiotherapy	First diagnosis of vaginal recurrence
(a) ISBT with EBRT										
1	41	Cervical	IIB	SCC	U	24	18	9.4	No	Yes
2	45	Cervical	IB1	SCC	U	44	30	14.2	No	Yes
3	58	Endometrial	IA	AD	U	29	22	25.6	No	Yes
4	69	Endometrial	IA	AD	U	15	11	30	No	No*
(b) ISBT alone										
5	54	Endometrial	IA	AD	E	31	21	24.4	No	Yes
6	57	Endometrial	IA	AD	U	20	7	8.2	No	Yes
7	57	Endometrial	II	AD	E	32	14	55.7	No	Yes
8	87	Endometrial	IB	AD	E	17	12	17.9	No	Yes
9	60	Cervical	IIB	AD	L	11	9	28.2	No	No*
10	58	Cervical	IIB	SCC	U	12	7	12.9	Yes (50 Gy/25fr)	Yes
11	78	Cervical	IB2	SCC	U	10	5	172.3	Yes (45 Gy/25fr)	Yes

*ISBT* interstitial brachytherapy, *EBRT* external beam radiotherapy, *FIGO* International Federation of Gynecology and Obstetrics 2008, *SCC* squamous cell carcinoma, *AD* adenocarcinoma, *U* upper one-third of the vagina, *L* lower one-third of the vagina, *E* upper two-thirds of, or the entire, vagina, *fr* fraction

^*^This was the second vaginal recurrence in these two patients; the first recurrences were treated with excision and chemotherapy

The treatment characteristics are summarized in Table 2. Three patients received concurrent chemotherapy. Weekly cisplatin was administered to two patients with squamous cell carcinoma, and tri-weekly cisplatin plus weekly paclitaxel was administered to one patient with adenocarcinoma. The median EQD2 received by at least 90% of the CTV (CTV D90) for all treatments was 73.6 Gy (range 50.8–98.2 Gy). The median CTV D90 for ISBT with EBRT was 82.2 Gy (range 60.4–84.2 Gy), and that for ISBT alone was 69.0 Gy (range 50.8–98.2 Gy). The median EQD2 received by a 2 cc area of the rectum or bladder for all treatments was 51.0 Gy (range 18.3–79.9 Gy) or 69.9 Gy (range 37.2–85.6 Gy), respectively.

**Table 2:** Treatment characteristics and clinical outcomes of each radiotherapy method

Treatment characteristics							Clinical outcomes		
Patient no	Current treatment method	Prescription dose	CTV		D2cc (Gy)		Local recurrence	Any recurrence	Late treatment-related toxicity
			Volume of BT (cc)	D90 (Gy)	Rectum	Bladder			
(a) ISBT with EBRT									
1	CRT (CDDP)	ISBT 30 Gy/5fr + EBRT 50 Gy/25fr	36	84.2	66.6	75.2	No	Liver, abdominal lymph node	No
2	CRT (CDDP)	ISBT 30 Gy/5fr + EBRT 50 Gy/25fr	49	83.0	73.0	80.7	No	No	GI(grade 1)
3	CRT (TP)	ISBT 30 Gy/5fr + EBRT 50 Gy/25fr	18	60.4	64.2	69.9	No	No	No
4	RT	ISBT 30 Gy/5fr + EBRT 50 Gy/25fr	34	81.5	69.6	78.3	No	Vagina*, inguinal lymph node, lung	GI(grade 1), GU(grade 3)
(b) ISBT alone									
5	RT	ISBT 42 Gy/7fr	47	98.2	24.3	85.6	No	Lung	GU(grade 1), pubic bone fracture(grade 1)
6	RT	ISBT 36 Gy/6fr	22	50.8	18.3	45.6	No	No	No
7	RT	ISBT 42 Gy/7fr	35	94.6	79.9	73.5	No	No	GI(grade 2)
8	RT	ISBT 42 Gy/7fr	34	73.6	40.8	56.0	No	No	No
9	RT	ISBT 42 Gy/7fr	32	69.0	39.7	37.2	No	Lung	Lymphoedema(grade 1)
10	RT	ISBT 36 Gy/6fr	23	63.7	29.9	38.6	No	No	No
11	RT	ISBT 42 Gy/7fr	28	68.0	51.0	53.9	No	No	GI(grade 3), GU(grade 1)

*ISBT* interstitial brachytherapy, *EBRT* external beam radiotherapy, *CRT* chemoradiotherapy, *RT* radiotherapy, *CDDP* cisplatin, *TP* cisplatin plus paclitaxel, *CTV* clinical target volume, *BT* brachytherapy, *CTV D90* the equivalent dose in 2 Gy fractions (EQD2) received by at least 90% of the CTV, *D2cc* the median EQD2 received by a 2 cc area, *fr* fraction, *GU* genitourinary, *GI* gastrointestinal

*The new vaginal recurrence was developed outside of the irradiated area and was treated by ISBT again; however, after 3 months, she developed additional metastases

**Fig. 1:**
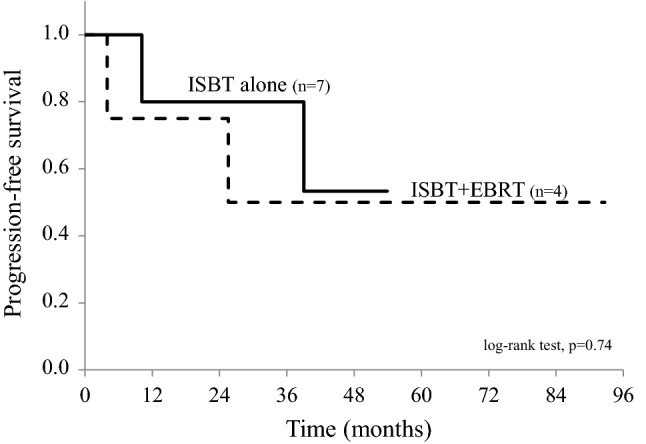
Progression-free survival for each radiotherapy method. Among the 11 patients, 4 of whom were treated with image-guided interstitial brachytherapy (ISBT) plus external beam radiotherapy (EBRT) and 7 with ISBT alone, clinical failure was observed in 4 patients (36.4%). Of these, two patients were treated with ISBT plus EBRT and the other two with ISBT alone. The 2-year progression-free survival (PFS) rate was 75.0% in the patients treated with ISBT plus EBRT and 80.0% in the patients treated with ISBT alone; the difference was not significant (log-rank test, *p* = 0.74)

The median follow-up period from the completion of radiotherapy was 43.9 months (range 3.9–92.7 months). The clinical outcomes are summarized in Table 2. At the final follow-up, all patients were alive except one, who died of a cerebral hemorrhage, but clinical failure was observed in four patients (36.4%). Local recurrence did not occur in any patient, and thus the 2-year LC rate was 100%. The median time to any recurrence was 17.9 months (range 3.9–39.0 months). The 2- and 4-year PFS rates of all patients were 78.8% and 52.5%, respectively. Of the four patients with clinical failure, two were treated with ISBT plus EBRT and two with ISBT alone. One patient treated with ISBT plus EBRT developed a new second vaginal recurrence and was treated with ISBT again because the recurrence was located outside of the irradiated area; however, after 3 months, she developed additional metastases in the inguinal lymph nodes and lung. The other three patients experienced distant metastasis only, occurring in the inguinal lymph nodes, abdominal lymph nodes, liver, and lung. The 2-year PFS rate was 75.0% for the patients treated with ISBT plus EBRT and 80.0% for the patients treated with ISBT alone; however, the difference was not significant (*p* = 0.74, Fig. 1). The 2- and 4-year vaginal recurrence-free survival rates of all patients were 100% and 87.5%, respectively.

Regarding treatment-related toxicities (Table 2), no acute toxicities of grade 3 or higher were observed. Two patients (18%) experienced late toxicities of grade 3. The one patient with the grade 3 gastrointestinal toxicity had been treated with pelvic EBRT prior to vaginal recurrence. The other patient with grade 3 genitourinary toxicity was treated with radiotherapy twice for two cases of vaginal recurrence.

## Discussion

Radiotherapy is a definitive treatment method for vaginal recurrence without other metastases, and BT is critical for this treatment. The HDR-BT dose can be calculated using CT or MRI, which is suggested to maximize disease control and reduce toxicity [4]. We present our initial experience with image-guided ISBT with or without EBRT for treatment of vaginal recurrence.

In the present study, the 2-year LC rate was 100%, and the 2- and 4-year vaginal recurrence-free survival rates were 100% and 87.5%, respectively. All 11 patients had received CT-based image-guided ISBT with (*n* = 4) or without (*n* = 7) EBRT, and the median CTV D90 was 73.6 Gy (range, 50.8–98.2 Gy). The Japanese Radiation Oncology Study Group [5] reported a 5-year LC rate of 83% after definitive radiotherapy or chemoradiotherapy in vaginal cancer patients from ten Japanese institutes. Among patients with vaginal recurrence of both cervical and endometrial cancers, 3-year LC rates of 78% and 85% were reported by Yoshida et al. and Kotsuma et al., respectively [3, 6]. Jhingran et al. [7] reported 2- and 5-year LC rates of 82% and 75%, respectively, after definitive radiotherapy for isolated vaginal recurrence of endometrial cancer. In a literature review, the American Brachytherapy Society (ABS) [1] reported 2- to 5-year LC rates of 83–100% in patients with vaginal recurrence of endometrial cancer. That review included three studies of patients treated with image-guided BT, which reported 2-year LC rates of 96% (CTVD90: 74.8 Gy) and 92% (CTVD90: 83 Gy) and a 3-year LC rate of 95% (CTVD90: 76 Gy). The CTV D90 and LC rate in the present study were consistent with those studies, despite our low patient number and heterogeneous patient population.

Two late toxicities of grade 3 were reported in the present study. The patient who developed grade 3 gastrointestinal toxicity had received previous pelvic radiotherapy, at a dose of 45 Gy in 25 fractions. She was treated with ISBT alone using a tumoricidal radiation dose, and the total EQD2 received by a 2 cc area of the rectum by both radiotherapy treatments was 94.2 Gy. Another patient who developed grade 3 genitourinary toxicity had been treated with radical radiotherapy twice for two cases of vaginal recurrence. In the ABS review of patients with vaginal recurrence of endometrial cancer previously treated with radiotherapy, a > 10% risk of developing grade 3 toxicities was reported [1]. Therefore, the two cases of grade 3 toxicity in our study do not appear to be unusual. The LC rate was 100%, which is consistent with previous studies, and no grade 3 late treatment-related toxicities developed in the eight patients who received radiotherapy only once. This result is suggested to be an advantage of image-guided ISBT.

According to the ABS review [1], EBRT at a 45–50 Gy dose should be administered to the pelvis before BT if the patient has received no previous pelvic radiation, to treat the lymph nodes and paravaginal tissues and shrink the gross disease in the vaginal cuff so that BT can be used to treat a smaller volume. Baek et al. [8] reported that none of 17 patients who received EBRT combined with BT, which included 2D planning BT, for vaginal recurrence of endometrial cancer experienced nodal recurrence, whereas 6 of 26 patients (23%) who received BT alone experienced nodal recurrence (*p* = 0.047). In the study by Jhingran et al. [7] involving 91 patients with vaginal recurrence of endometrial cancer treated with a combination of EBRT and BT (57%), EBRT alone (31%), or BT alone (12%), the LC rate after the EBRT and BT combination was significantly better compared with EBRT alone (*p* = 0.003) and tended to be better compared with BT alone (*p* = 0.09). Those authors reported that most of the recurrence cases were distant metastasis alone (*n* = 23), whereas pelvic wall recurrence was detected in only 1 of 43 patients with a second recurrence. Among our patients with vaginal recurrence of both cervical and endometrial cancers, there were no cases of pelvic area or local recurrence following treatment, although seven patients were treated with image-guided ISBT alone, of whom five had not received previous pelvic radiotherapy. There was one case of regional recurrence in the vagina, in a patient treated with the EBRT and ISBT combination, and this site was located along the external side of the urethral opening, outside of the irradiated area. Kotsuma et al. [6] also reported a 3-year LC rate of 78%, with no cases of nodal failure without local recurrence, among 14 patients, including 7 treated with image-guided ISBT alone and 7 treated with both EBRT and ISBT. One patient experienced nodal failure, as well as distant metastasis. Additionally, in the present study, the radiotherapy method (ISBT with EBRT vs. ISBT alone) had no significant effect on the PFS rate (*p* = 0.74). Thus, our image-guided ISBT regimen (ISBT with or without EBRT) was suggested to be appropriate. EBRT was given, before ISBT, when the recurrent tumor was suspected to involve microscopic invasion to paravaginal tissues or was too large to treat with ISBT alone. ISBT alone has the advantage of fewer treatment days compared with ISBT plus EBRT. Then, considering the site and size of the recurrent vaginal tumor, by not using EBRT in all cases, some patients had the benefit of a shorter treatment period.

The one case of vaginal recurrence among the 11 patients (9%) in the present study was defined as a second vaginal recurrence. In the Postoperative Radiation Therapy after Endometrial Cancer (PORTEC) trial [9], 2 out of 35 patients (6%) developed a second vaginal recurrence after curative treatment for the initial isolated vaginal recurrence of endometrial cancer. Sorbe et al. [10] also reported that 5 out of 40 patients (12%) developed a second vaginal recurrence after BT with or without EBRT, and one patient (3%) developed a third vaginal recurrence. The rate of development of the second vaginal recurrence in the present study was consistent with those studies. Additionally, the one case of a second vaginal recurrence was included in our calculations of the 2- and 4-year vaginal recurrence-free survival rates (100% and 87.5%, respectively), which are consistent with the LC rates of previous studies.

In conclusion, our image-guided ISBT regimen (comprising ISBT with or without EBRT) for postsurgical vaginal recurrence of cervical and endometrial cancers yielded effective treatment outcomes.
